# Coronavirus Disease 2019 Pandemic as Catalyst for Telemedicine Adoption: A Single-Center Experience

**DOI:** 10.1089/tmr.2020.0003

**Published:** 2020-11-18

**Authors:** Kunal Malhotra, Aparna Sivaraman, Hariharan Regunath

**Affiliations:** ^1^ Department of Medicine, University of Missouri System, Columbia, Missouri, USA.; ^3^ Department of Medicine, Division of Pulmonary & Critical Care & Infectious Disease, University of Missouri System, Columbia, Missouri, USA.; ^2^ Rock Bridge High School, Columbia, Missouri, USA.

**Keywords:** ambulatory care, COVID-19, SARS CoV-2, telemedicine

## Abstract

**Background:** Telemedicine use has increased due to stay-at-home orders during the novel coronavirus 2019 pandemic. We explored ambulatory provider's perception on telemedicine.

**Methods:** An anonymized survey was e-mailed to physicians and midlevel providers of our university hospital ambulatory clinics to assess current use, preferences for future use, and satisfaction with televisits.

**Results:** Of all providers, 249 responded (response rate 24%, 121 [48.6%] men, 177 [71.1%] attending physicians, 43 [17.2%] trainees, and 29 [11.6%] midlevel providers). Most respondents (120, 48.2%) belonged to subspecialties in medicine. At the time of the survey, 168 (67.5%) were using telemedicine for less than half of all visits and had video capabilities, of whom 224 (90%) considered it to be effective for return visits and 37 (15%) perceived it to be effective for new patients, 217 (87.1%) wanted to continue with telemedicine practice, and 113 (45.4%) preferred to use telemedicine for more than a quarter of their future patients even after the pandemic. Most (194 [77.9%]) were satisfied with telemedicine and we found no differences among specialties. Those with audio-only visits reported least effectiveness for new patient evaluation (*p* < 0.001) and overall provider satisfaction (*p* = 0.02) when compared with others. Those who saw more than a quarter of their patients through televisits desired to increase their future televisits to >50% including new patients (*p* < 0.001).

**Conclusions:** There is widespread interest in telemedicine in all specialties. Acceptance is high for return visits, but low for new patient visits. Improvement in technology to have both audio and video capability consistently may foster further interest toward increasing telemedicine in the future.

## Introduction

The coronavirus disease 2019 (COVID-19) has created an imbalance between many service industries and consumers including health care. As the number of cases continue to increase over time, telehealth offers an ideal platform because care delivery becomes possible without risks of exposure to crowded environments.^1–3^ Although the advantages of telehealth are well known, it was still underutilized for a variety of reasons, including clinician acceptance, issues in credentialing, insurance/reimbursement, workflow, and access to telehealth and infrastructure.^4–7^ We are now seeing an adaptive rise in the use of telehealth to provide consultations remotely to obviate the need for physical distancing and personal protective equipment.^2^,^3^,^8^

Our University health care system has 58 ambulatory clinics, of which 42 are on-site, 11 within 7 miles (11,265 meters) from the main university hospital, 5 in neighboring towns within 40 miles (64,373 m), and additional 3 outreach clinics within a 70-mile (112,654 m) radius from the university hospital. There were a total of 738,749 outpatient visits in the financial year 2020 (i.e., July 2019–June 2020). Before the pandemic, there was low adoption to telehealth due to lack of provider engagement and/or reimbursement). The outreach clinics in child psychiatry and dermatology were the primary locations for telehealth, mainly located in the rural part of our service area. In 2017, we began offering virtual urgent care services directly to consumers, using the American Well platform. Before the COVID-19 pandemic, very few of our on-site ambulatory clinics conducted scheduled telehealth visits.

After the start of this pandemic, we quickly implemented telehealth within our ambulatory clinics utilizing Zoom (Copyright © Zoom Video Communications Inc.) technology. Our institution's providers already had an existing Zoom account that facilitated rapid enabling of a Health Insurance Portability and Accountability Act (HIPAA) compliant version. Zoom also allowed multiple care team members to participate in a patient's care (nurses, midlevel providers, medical students, residents, fellows, and attending physicians). We also continued to use American Well for on-demand urgent care visits, where patients could self-select a provider to see them for low-acuity care in Emergency Medicine, Quick Care Clinics, and the American Online Care Group (provider network). Providers were given training for using it along with the best practice and documentation guidelines. Nurses and clinic appointment schedulers also underwent training to assist patients with their appointments. Given the quick implementation of telehealth visits, we performed a survey to evaluate our provider's perception of telehealth in relation to their current and future ambulatory practice.

## Methods

An anonymized survey (see Supplementary Appendix) was e-mailed to all ambulatory health care providers within the School of Medicine at University of Missouri, after approval from the institutional review board (IRB) during April–May, 2020 (IRB No. 2022646). The anonymous survey included 10 closed-ended questions with predetermined answers as options. They assessed the use of telemedicine among out-patient providers, their preferences for current and future practice, the type of patient visits, etc. There were no open-ended questions and we avoided the use of judgmental, negative, or double-barreled statements.^9^ Face validity was established by subjective assessment of the survey questions by a group of 12 attending physicians within the hospital leadership, 6 resident physicians, and 15 fellows. This group assessed feasibility, readability, consistency in style, format, language, and deemed it to be appropriate for this study's purpose and content area.

The target population included attending physicians, fellows, residents, and midlevel providers who have out-patient (ambulatory) clinics within University of Missouri Health Care. They were identified through specific membership lists (group e-mail accounts) within the University of Missouri e-mail system. Our e-mail stated that their participation is voluntary and there will not be any monetary or other incentives. Design, distribution, and data collection for this article were generated using Qualtrics^XM^ (© 2020 Qualtrics^®^, Provo, UT). Sampling frame data were imported into and analyzed using SPSS Version 25.0 statistic software package for Windows and Microsoft Excel 2019.

Demographic and descriptive data are represented as numbers and percentages, chi-squared test was used for comparison of proportions between type of telemedicine use and the respondent's perceived levels of satisfaction and future telehealth use.

## Results

Out of 1032 e-mail recipients in the sampling frame, 249 completed all questions (24.1% response rate, men 121 [48.6%]) and most of them were attending physicians (177, 71.1%). Demographics of all responders are given in Table 1.

**Table 1.** Demographics

	Frequency	%
Gender		
Men	121	48.6
Women	48	19.3
No answer	8	3.2
Type of provider		
Attending	177	71.1
Fellow	15	6.0
Resident	28	11.2
NP/PA	29	11.6
Specialty		
Medicine	120	48.2
Surgery	31	12.4
Family medicine	19	7.6
Child health	16	6.4
OBGYN	14	5.6
Psychiatry	12	4.8
Ortho	9	3.6
Neuro	8	3.2
Radiology	7	2.8
PM&R	7	2.8
Dermatology	3	1.2
Ophthalmology	3	1.2

NP, nurse practitioner; OBGYN, obstetrics and gynecology; PA, physician assistant, PM&R, physical medicine and rehabilitation.

**FIG. 1.**
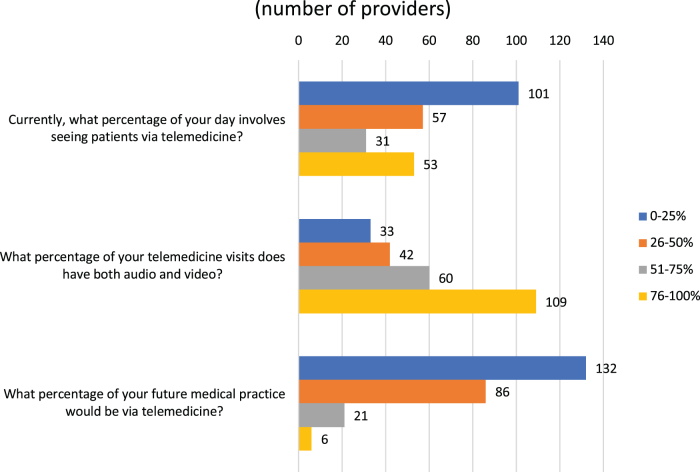
Responses to telemedicine practice questions at present and in future.

At the time of the survey, 93.2% (232/249) had already used telehealth since the start of the COVID-19 pandemic. Given the low usage of telemedicine before the pandemic, most users were new to telehealth and were doing it for the first time. Figure 1 shows telemedicine usage across all physicians. Most providers were practicing telemedicine <50% of the day (168, 67.5%) and had both audio and video components. Table 2 shows that although majority (224, 90%) believed that telemedicine was effective for return patients, only 14.5% (36/249) thought that it will be effective for >75% of their new patients. Satisfaction was high (195, 78.3%) and most (212, 85.1%) of the providers wanted to continue telemedicine. Of this, 45.4% (113/249) said they wanted more than at least 25% of their future patient visits as telehealth even after the COVID-19 pandemic.

**Table 2.** Responses to Questions

	n (%)		n (%)
In what percentage of new patients is telemedicine an effective way of delivering health care?		How satisfied are you delivering health care through telemedicine?	
0–25%	73 (29.3)	Extremely dissatisfied	4 (1.6)
26–50%	72 (28.9)	Somewhat dissatisfied	19 (7.6)
51–75%	62 (24.9)	Neither dissatisfied or satisfied	26 (10.4)
76–100%	36 (14.5)	Somewhat satisfied	114 (45.8)
*n*/a	6 (2.4)	Extremely satisfied	81 (32.5)
Do you feel telemedicine is an effective way of delivering health care to return patients?		Will you keep practicing telemedicine after COVID-19 pandemic gets under control?	
No	17 (6.8)	No	32 (12.9)
Yes	224 (90.0)	Yes	212 (85)
*n*/a	8 (3.2)	*n*/a	5 (2)

COVID-19, coronavirus disease 2019; n/a, no answer.

**FIG. 2.**
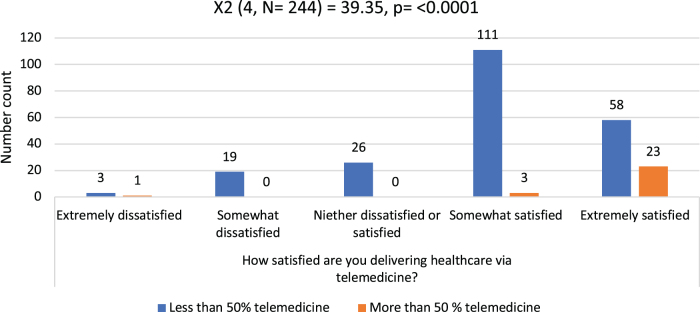
Future telemedicine use based on current satisfaction.

**FIG. 3.**
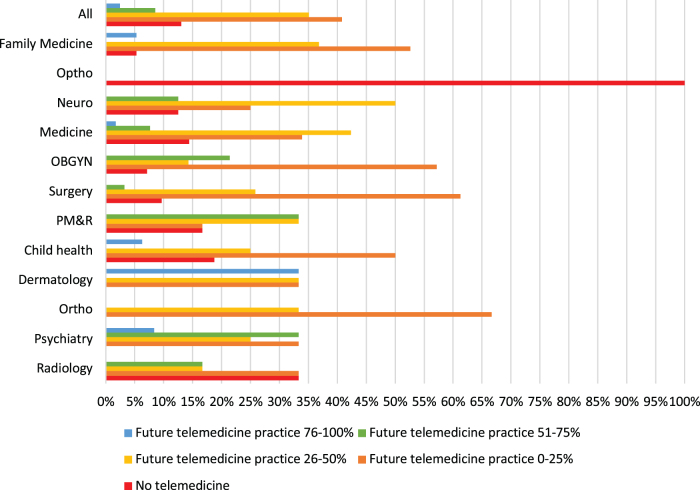
Future telemedicine use by specialty. OBGYN, obstetrics and gynecology; PM&R, physical medicine and rehabilitation.

There was no difference in the proportion of satisfied providers between surgical and nonsurgical specialties (*χ^2^* = 5.46 [*df* = 4, *N* = 244], *p* = 0.243) and gender (*χ^2^* = 7.19 [*df* = 8, *N* = 244], *p* = 0.516). However, those who mostly did audio visits only perceived the least effectiveness of telemedicine for new patient evaluations (*χ^2^* = 14.78 [*df* = 1, *N* = 241], *p* < 0.001) when compared with others. Overall satisfaction with telemedicine was also significantly reduced in the same group (*χ^2^* = 9.48 [*df* = 1, *N* = 242], *p* = 0.02), that is, “somewhat dissatisfied” or “extremely dissatisfied,” when compared with others. Figure 2 compares providers with different levels of satisfaction to see whether that affects their desire to do telemedicine in future. Those who perceived televisits to be effective for new patient visits wanted more than half of their future practice as televisits (*χ^2^* = 7.26 [*df* = 1, *N* = 242], *p* = 0.007) than others. Similarly, those who were somewhat or extremely satisfied with telemedicine also expressed a significantly higher desire to do >50% of their future practice as telemedicine (Fig. 2). Specialty-wise preferences for telemedicine use in their future practice are depicted in Figure 3, of whom 32 (13%) providers did not want to do any telemedicine in their future practice, the rest (217, 87%) wanted to do at least some of their future practice as telemedicine.

## Discussion

Telemedicine existed long before the COVID-19 pandemic, however, the current pandemic has evolved as an important milestone for telemedicine due to its rapid adoption and implementation seen never before. This was largely due to elimination of reimbursement barriers and emergency policy changes. In March 2020, U.S. Centers for Medicare and Medicaid Services (CMS) broadened the access of telemedicine services removing geographical barriers to care for patients. It allowed reimbursements for office, hospital, and other visits furnished through telehealth across the country and including patient's places of residence.^10^ Further changes in April 2020 allowed physicians to care for patients across state lines, through phone, radio, or online communications and temporarily waived certain training, certification, regulatory, and paperwork requirements.^11^ In contrast, the patients' acceptance also increased, as demonstrated by a survey of 2000 Americans in March 2020, which reported ∼60% of their respondents were more likely to consider telemedicine services for the future.^12^

The American College of Physicians surveyed a random sample of 1972 physician members in the United States aged 65 years and younger between December 2019 and January 2020. Of the 231 respondents (11.7% response rate) providing patient care (49% general internal medicine, 24% hospital medicine, and 26% subspecialists), primary barriers to telemedicine adoption were financial and structural concerns, but not lack of interest.^13^ Before the COVID-19 pandemic, obstacles from insurers and the federal government prevented physicians from fully embracing the complete potential of telehealth. In addition, at the time of this survey, telephone call visits were not covered by any insurance payers, so they were not considered as billable visits and thus was not measured in this survey. However, during the pandemic, CMS and many other payers started paying for audio-only telephone visits.^13,14^

Provider reluctance, technical challenges for clinic staff, cost, reimbursement issues, lack of interest, patient's age, and level of education were some of the major barriers to telemedicine in the pre-COVID-19 era.^4–7^ From our results, it is evident that providers have adapted and embraced telemedicine and that the pandemic-related changes in health policies have removed most of these barriers. Given the widespread adoption and convenience, the demand on telemedicine will continue to rise in the future. HIPAA-compliant resources to improve broadband access, remote monitoring, and integration into electronic medical records without compromising quality are important. Physicians and patients will probably continue to have the choice of selecting between telemedicine or in-person visits, and between video or audio/telephone-only visits. Health care systems will have to leverage into this diversity to meet their patient care needs, as there will still be a few providers and patients who would rather prefer in-person visits only. Training the staff, nurses, and other health care workers on ethics and best practices of telemedicine is important for appropriate billing and documentation nuances. Current medical school curriculum does not incorporate education on telemedicine, and given the surge on use of telemedicine, it becomes important to consider its inclusion as COVID-19 is not anticipated to end soon, but rather prevail into our future. This is further reinforced by most U.S. residency and fellowship programs that have already incorporated televisits in their ambulatory environment.

Another finding in our study is that there is widespread interest in telemedicine even among surgical specialties. Of the three ophthalmologists, none expressed interest in telemedicine and this reflects the nature of their specialty wherein there is a need for examining the eye and current technology does not support it. Having audio and video capability leads to a higher satisfaction, hence having both is important to foster further interest toward increasing telemedicine in their future practice. Growth in broadband^15^ and use of video-capable smart phones^16^ have been steadily increasing over the years and will fuel continued growth of telemedicine.

Limitations include a single-center study in an academic setting, low response rate compared with the sampling frame, less respondents from nonmedical/surgical subspecialties, and the use of face validity. Multicenter studies using an objectively validated questionnaire including nonacademic settings will provide further insights.

### Conclusions and Future Perspectives

Telemedicine is here to stay and its growth will continue. It has been rapidly adopted by all key stakeholders, that is, patients, payers, health care organizations, and providers. Providers will use it more in future and its effectiveness depends on the sustenance and future evolution of technology, health outcomes, and satisfaction among participants. We need better training, and infrastructure including both audio–video capabilities and flexibility to adapt to varying needs. Solutions would have to be customized to the needs of individual health care organizations or practice setting and specialty.

## Supplementary Material

Supplemental data

## Abbreviations Used

CMS: Centers for Medicare and Medicaid Services
COVID-19: coronavirus disease 2019
HIPAA: Health Insurance Portability and Accountability Act
IRB: institutional review board
