# Supplementary material for: Coronavirus Disease 2019 Pandemic as Catalyst for Telemedicine Adoption: A Single-Center Experience
Source: Telemed Rep. 2020 Nov 18;1(1):16–21. doi: 10.1089/tmr.2020.0003 (PMC8812292; doi:10.1089/tmr.2020.0003)
Supplement: Supplemental data [file Supp_Data.docx]

Assessment of use of Telemedicine in an outpatient academic center during COVID-19 Pandemic.

Q1 Have you seen patients via telemedicine before COVID-19 pandemic?

- Yes (1)
- no (2)

Q2 Are you currently seeing patients via telemedicine?

- Yes (1)
- No (2)

Q3 What is your gender ?

- Male (1)
- Female (2)

Q4 Currently, what percentage of your day involves seeing patients via telemedicine?

- 0-25% (1)
- 26-50% (2)
- 51-75% (3)
- 76-100% (4)

Q5 What percentage of your telemedicine visits does have both audio and video?

- 0-25% (1)
- 26-50% (2)
- 51-75% (3)
- 76-100% (4)

Q6 In what percent of new patients  telemedicine is an effective way of delivering healthcare ?

- 0-25% (1)
- 26-50% (2)
- 51-75% (3)
- 76-100% (4)

Q7 Do you feel  telemedicine  is an effective way of delivering healthcare to return patients?

- Yes (1)
- No (2)

Q8 How satisfied are you delivering healthcare via telemedicine?

- Extremely satisfied (1)
- Somewhat satisfied (2)
- Neither satisfied nor dissatisfied (3)
- Somewhat dissatisfied (4)
- Extremely dissatisfied (5)

Q9 1.       Will you keep practicing telemedicine after COVID-19 pandemic gets under control?

- Yes (1)
- No (2)

Q10 What percentage of your future medical practice would be via telemedicine?

- 0-25% (1)
- 26-50% (2)
- 51-75% (3)
- 76-100% (4)

End of Block: Default Question Block
